# Survey of legislative frameworks and national recommendations governing paediatric maintenance haemodialysis in Europe

**DOI:** 10.1007/s00467-025-06667-8

**Published:** 2025-01-23

**Authors:** Enzo Vedrine, Claus Peter Schmitt, Johan Vande Walle, Diamant Shtiza, Klaus Arbeiter, Evelien Snauwaert, Danka Pokrajac, Dimitar Roussinov, Danko Milosevic, Elia Avraam, Jakub Zieg, Ida Maria Schmidt, Ylle Toots, Tuula Holtta, Günter Klaus, Varvara Askiti, Kalman Tory, Clodagh Sweeney, Enrico Verrina, Edite Jeruma, Augustina Jankauskiene, Valerie Said Conti, Branko Lutovac, Linda Koster-Kamphuis, Velibor Tasic, Anna Kristina Bjerre, Maria Szczepańska, Alberto Caldas Afonso, Andreea Liana Rãchişan, Brankica Spasojevic, Victor Janko, Gregor Novljan, Pedro J. Ortega, Lisa Sartz, Sibylle Tschumi, Sevcan Azime Bakkaloglu, Jan Dudley, Dymtro D. Ivanov, Rukshana Shroff, Bruno Ranchin, with the support of the EPDWG, ERKNet and ESPN dialysis working groups

**Affiliations:** 1 Centre de Référence des Maladies Rénales Rares, Hôpital Femme Mère Enfant, Hospices Civils de Lyon, 59 boulevard Pinel, 69677 Bron Cedex, France; 2 Center for Pediatric and Adolescent Medicine, University Hospital Heidelberg, Heidelberg, Germany; 3 Department of Pediatric Nephrology, Ghent University Hospital, Ghent, Belgium; 4 Department of Pediatric Nephrology “Mother Teresa” Hospital – Tirana, Tirana, Albania; 5 Department of Pediatric and Adolescent Medicine, Medical University Vienna, Vienna, Austria; 6 Department of Pediatric Nephrology, Ghent University Hospital, Ghent, Belgium; 7 Pediatric Clinic, Clinical Centre University of Sarajevo, Sarajevo, Bosnia and Herzegovina; 8 SBAL Pediatric Diseases, Nephrology and Hemodialysis Clinic, Department of Pediatrics, Medical University of Sofia, 1606 Sofia, Bulgaria; 9 University of Zagreb School of Medicine, University Hospital Center Zagreb, Zagreb, Croatia; 10 Department of Pediatrics, Archbishop Makarios III Hospital, Nicosia, Cyprus; 11 Department of Pediatrics, 2nd Faculty of Medicine, Charles University in Prague and Motol University Hospital, V Úvalu 84, 15006 Praha 5, Czech Republic; 12 Department of Pediatrics and Adolescent Medicine, University Hospital Rigshospitalet, Copenhagen, Denmark; 13 Department of Pediatric Tallinn Children’s Hospital, Tallinn, Estonia; 14 Department of Pediatric Nephrology and Transplantation, The New Children’s Hospital, HUS Helsinki University Hospital, Helsinki, Finland; 15 KfH Pediatric Kidney Center and Department of Pediatrics, Philipps University, Marburg, Germany; 16 Department of Nephrology, “P. and A. Kyriakou” Children’s Hospital, Athens, Greece; 17 Pediatric Centre, MTA Centre of Excellence, Semmelweis University, Budapest, Hungary; 18 National Pediatric Hemodialysis Centre and Renal Transplant Unit, Temple Street Children’s University Hospital, Dublin, Ireland; 19 Division of Nephrology, Dialysis, and Transplantation, Instituto di ricovero e cura a carattere scientifico (IRRCS) Instituto Giannina Gaslini Children’s Hospital, Genoa, Italy; 20 Pauls Stradiņš Clinical University Hospital, Pilsoņu iela 13, Zemgales priekšpilsēta, Rīga, LV-1002 Latvia; 21 Vilnius University, Children Hospital Affiliate of Vilnius University Hospital “Santariskiu klinikos”, Santariskiu 4, 08406 Vilnius, LT Lithuania; 22 Department of Pediatrics, Mater Dei Hospital Malta, Msida, Malta; 23 Institute for Children’s Diseases, Clinical Center of Montenegro, Prodgorica, Montenegro; 24 Department of Pediatric Nephrology, Radboud University Medical Center, Radboud Institute for Molecular Life Sciences, Amalia Children’s Hospital, Nijmegen, the Netherlands; 25 Medical Faculty Skopje, University Children’s Hospital, Skopje, Macedonia; 26 Pediatric Nephrology, Oslo University Hospital, Rikshospitalet, Oslo, Norway; 27 Chair and Department of Pediatrics, Faculty of Medical Sciences in Zabrze, Medical University of Silesia, Katowice, Poland; 28 Centro Materno Infantil do Norte, Centro Hospitalar Universitario de Santo António, Porto, Portugal; 29 Department of Pediatrics II, University of Medicine & Pharmacy “Iuliu Hatieganu”, Cluj-Napoca, Romania; 30 Department of Nephrology, Dialysis and Transplantation, University Children’s Hospital, Belgrade, Serbia; 31 Medimpax, Bratislava, Slovakia; 32 Pediatric Nephrology Department, Children’s Hospital, University Medical Centre Ljubljana, Ljubljana, Slovenia; 33 Department of Pediatric Nephrology, Hospital Universitari La Fe, Valencia, Spain; 34 Department of Pediatrics, Skane University Hospital, Lund University, Lund, Sweden; 35 Department of Pediatrics, Pediatric Nephrology, Inselspital, University of Berne, Berne, Switzerland; 36 Division of Pediatric Nephrology, Department of Pediatrics, Gazi University Faculty of Medicine, Ankara, Turkey; 37 Department of Pediatric Nephrology, Bristol Children’s Hospital, Bristol, UK; 38 Department of Nephrology and Renal Replacement Therapy, Shupyk National Health Care University, Kiev, Ukraine; 39 UCL Great Ormond Street Hospital and Institute of Child Health, London, UK

**Keywords:** Child, Legal status, Maintenance haemodialysis, Recommendations, Survey

## Abstract

**Background:**

The application of international recommendations for paediatric maintenance haemodialysis (HD) could be strengthened by national laws or written recommendations. Our aim was therefore to describe the national rules governing paediatric maintenance HD in European countries.

**Methods:**

A national representative, approved by the president of each paediatric nephrology society, was contacted in all 42 European countries to complete two online questionnaires.

**Results:**

Answers were received from 36 countries. The population served by HD centres varies from 83,000 to 1,197,000 residents below 18 years of age and the estimated mean number of children on HD per centre from 0.2 to 13.5. The lowest age at which a child can be dialysed in an adult centre varies from 0 to 18 years. Laws or written national recommendations specifying: this age, the need for a paediatrician as part of medical team in mixed adult–paediatric centres, the minimum number of doctors per centre and the number of patients per nurse or nurse’s aide required during sessions exist in only 25, 22, 22, 44 and 8% of the countries, respectively. Similarly, dietitians, social workers, school service, psychologists and play specialists/youth workers are required by law or written national recommendations in 36, 28, 36, 31 and 14% of countries, respectively.

**Conclusion:**

Laws or written national recommendations for paediatric maintenance HD are rare in European countries and very heterogeneous when they exist. This calls for discussion among paediatric and adult nephrologists and health authorities on the organisation of safe and effective paediatric HD practices.

**Graphical abstract:**

**Figure:**
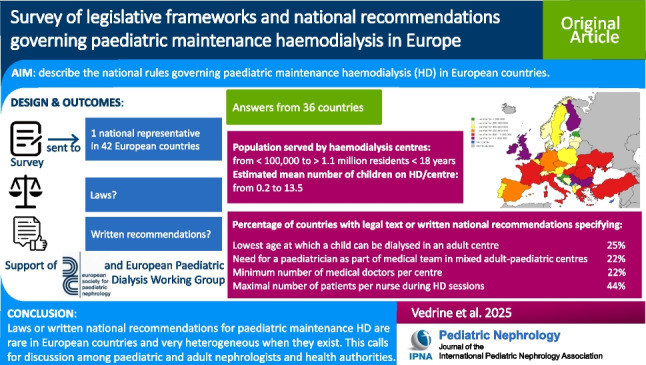
A higher resolution version of the Graphical abstract is available as Supplementary information

**Supplementary Information:**

The online version contains supplementary material available at 10.1007/s00467-025-06667-8.

## Introduction

Kidney transplantation is the best option for kidney replacement therapy (KRT), but many children require maintenance dialysis and nearly 50% of children requiring long-term dialysis in Europe are treated with haemodialysis (HD), as indicated by the most recent 2016 data from the ERA-EDTA registry [1]. Thus, about five children below 15 years of age per million are currently on maintenance HD in Europe [1], and the prevalence is likely to increase further since KRT has been widely accepted in younger children, those with complex multi-organ disease [1], and in otherwise stable children from the first days of life [2].

The principles of HD and the basic technical requirements are similar for adults and children, but the child’s size and the haemodynamic specifications require paediatric-specific adaptations of HD technology. Variations in body weight require extracorporeal system priming in the youngest children and precise ultrafiltration management. Within the paediatric HD population, children weighing less than 10 kg represent 2 to 9% of patients starting HD and those weighing less than 20 kg, 11 to 37% of this population [3, 4]. These small children are most challenging since they face a substantial lack of equipment adapted to their sizes and advanced dialysis techniques, such as online haemodiafiltration, blood volume monitoring, blood volume controlled ultrafiltration, access recirculation monitoring, temperature control, urea clearance monitoring and sodium control, have neither been designed nor validated for children, but healthcare staff working in paediatric dialysis centres often use such devices or options off label [3, 4].

Moreover, the children receiving HD suffer from a large variety of underlying diseases, each of which demands special knowledge and treatment, and these patients are particularly vulnerable to the long-term consequences of inadequate schooling and psychosocial support.

In this context, the European Paediatric Dialysis Working Group (EPDWG) and the American Society of Pediatric Nephrology have described the components of care necessary for children on dialysis therapy, advocating care by a multidisciplinary team in a paediatric unit [5, 6]. However, their application could be strengthened by national laws or written recommendations that address the minimal human and technical resources required for proper care and patient needs.

The aim of this study was therefore to describe the laws and national recommendations underpinning maintenance HD for children in Europe. The results could form the basis for the development of national standards and enable countries that do not have a sufficient framework to begin a process of discussion and quality improvement in order to obtain resources for the care of children receiving HD.

## Methods

This study was performed with the support of the European Society for Paediatric Nephrology (ESPN), European Rare Kidney Disease Network, EPDWG, and the presidents of each national paediatric nephrology society in Europe.

National representatives, nominated and/or approved by the president of each national paediatric nephrology society, were contacted in all 42 European countries to complete two online questionnaires, the first between January and April 2023 and the second between September 2023 and January 2024. The two questionnaires were compiled during online meetings with members of the EPDWG together with the national representatives. The first questionnaire consisted of a comprehensive list of 19 questions on the legislative framework for paediatric maintenance HD, as well as structure and team composition of centres in charge of paediatric maintenance HD (Supplementary Table 1). The second questionnaire sought further details on the type of rules governing the organisation of paediatric HD: written legal text from national governments, written recommendations from the national paediatric nephrology society, unwritten consensus within the national paediatric nephrology community or none (Supplementary Table 2). In addition, a supplementary question regarding reimbursement for HD and transport was sent by email in September 2024.

The number of HD centres in charge of paediatric dialysis in each country was reported relative to the population under 18 years of age according to the United Nations World Population Prospects 2022 [7]. The prevalence of maintenance HD was estimated using the ERA-EDTA registry (courtesy of Dr. Marjolein Bonthus) for all countries with available data and using Kuratorium für Dialyse und Nierentransplantation (KfH) data (courtesy of Prof. Günter Klaus) for Germany.

## Results

We received responses to both questionnaires from 36/42 countries; all European Union countries where paediatric maintenance HD is performed responded. The response from the Luxembourg representative was that paediatric maintenance HD was not performed in his country. We did not receive a response from two countries (Georgia and Iceland), and the representatives of further three countries (Belarus, Moldova and Russia) responded to the first questionnaire only and were excluded from the analysis.

### Population served by HD centres

**Fig. 1:**
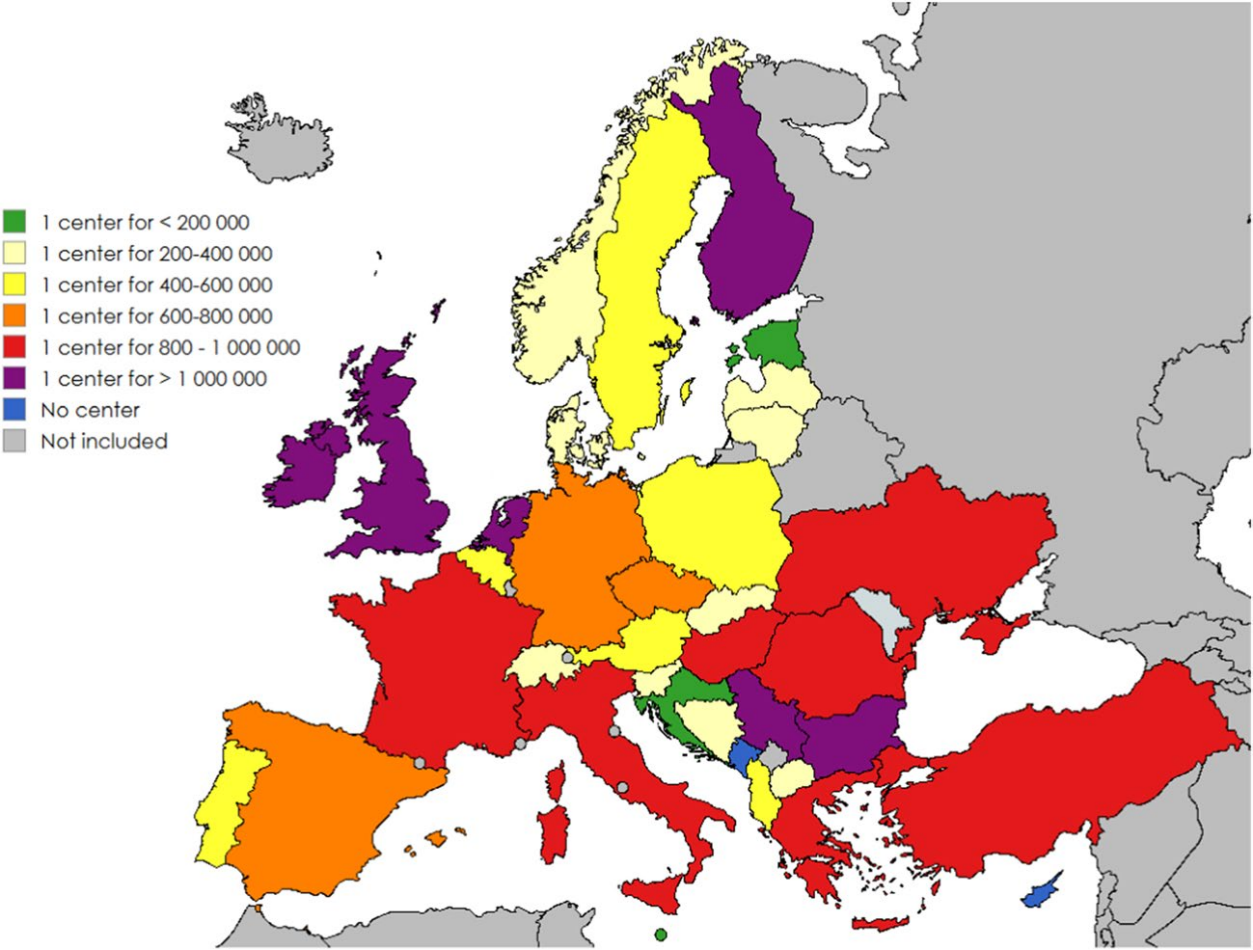
Number of paediatric residents (< 18 years of age) per dialysis centre providing paediatric maintenance haemodialysis in European countries

We identified 184 centres responsible for maintenance HD in children, of which 123 were exclusively paediatric HD centres and 61 were mixed adult–paediatric centres. The population density of centres that perform paediatric maintenance HD varies greatly: the representatives of two countries, Montenegro and Cyprus, state that there is no centre in their country. In the other countries, the number of centres in relation to the child population varies from one per 83,000 to one per 1,197,000 and the estimated mean number of children on HD per centre varies from 0.2 to 13.5 according to the prevalence of paediatric maintenance HD in each country (Table 1 and Fig. 1).

**Table 1:** Number of dialysis centres providing paediatric maintenance haemodialysis (HD), respective paediatric population below 18 years of age, prevalence of long-term HD, estimated mean number of children on HD per centre and staff requirements in European countries

Country	Number of centres	Number of centres exclusively paediatric	Residents of the country < 18 years	Paediatric residents of the country/centre	Prevalence of long-term HD, pmarp < 18 years^§^	Estimated mean number of children on HD/centre	Minimum number of MD per centre	Maximal number of patients during sessions	
								Per nurse	Per nurse’s aide
Albania	1	1	591,000	591,000	11.4*	6.74	NS	2	NS
Austria	3	1	1,562,000	520,667	1.8	0.94	NS	1	NS
Belgium	5	2	2,323,000	464,600	ND	ND	NS	NS	NS
Bosnia and Herzgovina	2	1	528,000	264,000	9.6	2.53	2	1	2
Bulgaria	1	1	1,191,000	1,191,000	2.5*	2.98	NS	NS	NS
Croatia	4	4	680,000	170,000	6.8*	1.16	2	2	4
Cyprus	0		253,000		4.2*		NS	NS	NS
Czechia	3	3	2,028,000	676,000	2.9*	1.96	1	NS	NS
Denmark	3	0	1,154,000	384,667	2.5	0.96	NS	1	NS
Estonia	2	2	263,000	131,500	3.8*	0.50	NS	NS	NS
Finland	1	1	1,033,000	1,033,000	1.8	1.86	NS	NS	NS
France	17	16	13,754,000	809,059	8.7	7.04	2	2	4
Germany	18	18	13,970,000	776,111	9.0^@^	6.99	1	NS	NS
Greece	2	2	1,760,000	880,000	8.9	7.83	2	3	3
Hungary	2	1	1,708,000	854,000	7.2*	6.15	2	3	NS
Ireland	1	1	1,197,000	1,197,000	3.2*	3.83	NS	3	NS
Italy	11	10	9,111,000	828,273	ND	ND	NS	3	NS
Latvia	1	0	359,000	359,000	2.7*	0.97	NS	4	NS
Lithuania	2	2	503,000	251,500	3.8*	0.96	1	3	NS
Malta	1	0	83,000	83,000	0.0*		NS	NS	NS
Montenegro	0		136,000		ND		NS	NS	NS
Netherlands	3	0	3,344,000	1,114,667	3.7	4.12	NS	NS	NS
North Macedonia	1	0	378,000	378,000	6.9*	2.61	1	4	4
Norway	5	0	1,110,000	222,000	0.9	0.20	NS	NS	NS
Poland	12	6	7,027,000	585,583	6.0*	3.51	1	NS	NS
Portugal	4	2	1,666,000	416,500	5.0*	2.08	NS	2	NS
Romania	4	4	3,721,000	930,250	14.5	13.49	1	4	NS
Serbia	1	1	1,179,000	1,179,000	5.2*	6.13	NS	3	NS
Slovakia	4	2	1,038,000	259,500	4.6*	1.19	1	4	4
Slovenia	1	1	379,000	379,000	7.5*^$^	2.84	NS	2	NS
Spain	11	8	8,007,000	727,909	4.1*	2.98	1	2	NS
Sweden	4	0	2,197,000	549,250	2.6	1.43	NS	NS	NS
Switzerland	4	4	1,580,000	395,000	2.4*^μ^	0.95	NS	NS	NS
Turkey	28	9	23,089,000	824,607	3.5*	2.89	1	NS	NS
Ukraine	9	7	7,325,000	813,889	6.0*	4.88	2	6	NS
UK	13	13	14,393,000	1,107,154	6.7^£^	7.42	3	3	NS

*pmarp* per million age-related population, *NS* not-specified, *MD* medical doctor, *ND* no data available

*Might be an underestimation of the true prevalence due to possible treatment in adult centres

^§^ERA EDTA registry data which refer to the prevalence on December 31, 2021, unless stated otherwise, courtesy from Marjolein Bonthuis

^@^Prevalence estimated using KfH (Kuratorium für Dialyse und Nierentransplantation) data, courtesy of Günter Klaus

^$^Prevalence data for the year 2020

^μ^Prevalence data for the year 2018

^£^Excluding patients from Scotland

### Reimbursement of maintenance HD and transport to centres

National health insurance covers the cost of paediatric maintenance HD in all studied countries, and transport to the centre is also fully covered by the national health insurance in all countries except Belgium, where some families have to pay part of the cost of transport.

### Regulations defining the minimum age for dialysis in an adult HD centre

**Fig. 2:**
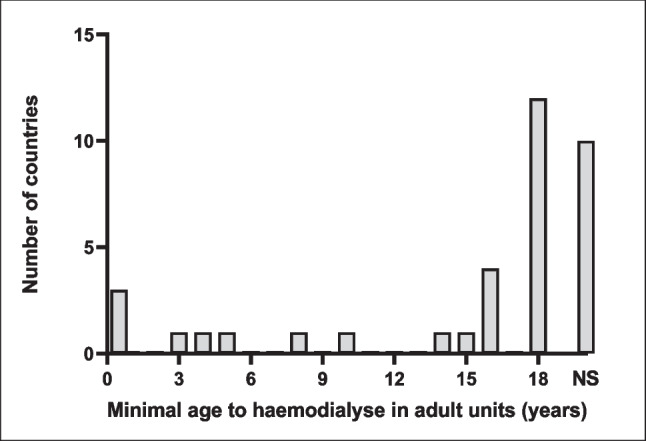
Minimal patient age required for maintenance haemodialysis in adult dialysis centres, in the 36 European countries

**Fig. 3:**
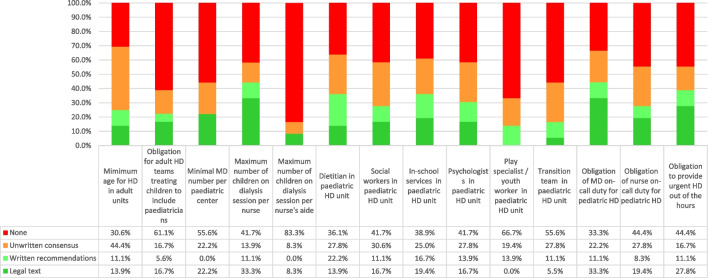
Type of rules governing maintenance paediatric haemodialysis (HD) in Europe: percentage of countries with each type of rules or without rules

The age at which a child can be dialysed in an adult HD centre is not specified in 10 (28%) countries. This age is highly variable in other countries, ranging from no lower limit in 3 (8%) to 18 years in 12 (33%). This lower age limit of 18 years is based on an unwritten consensus in 7 (19%) countries and on laws or written recommendations in 5 (14%). In 11 (31%) countries, children can be dialysed in adult centres from the age of 3 up to 16 years: 3 years in North Macedonia, 4 years in Estonia, 5 years in Ukraine, 8 years in France, 10 years in Lithuania, 14 years in Bosnia and Herzegovina, 15 years in Slovakia and 16 years in Greece, Ireland, Montenegro and the UK (Fig. 2). Overall this age is set by law in 5 (14%) countries and by written recommendations of paediatric nephrology societies in 4 (11%), while in the remaining 27 (75%) countries, only an unwritten national consensus or no rules were reported (Fig. 3 and Supplementary Table 3).

In nine (25%) countries, there are no dedicated paediatric dialysis centres, but seven (19%) of them provide mixed adult–paediatric centres (Table 1).

### Multidisciplinary teams in dialysis centres

The rules surrounding multidisciplinary teams caring for children in dialysis centres in charge of children are described in Table 1, Supplementary Table 3 and Fig. 3. Laws or written national recommendations regarding the minimal number of medical doctors per centre exist in 22% of countries, and these exist for the maximum number of patients per nurse during HD treatment in 44% countries and for the maximum number of patients per nurse aide in 8% (Table 1, Fig. 3 and Supplementary Table 3). The number of healthcare providers required varies considerably between these countries (Table 1).

Dietitians, social workers, school service, psychologists and play specialists/youth workers are required by law or written recommendations in 36, 28, 36, 31 and 14% of countries, respectively (Fig. 3 and Supplementary Table 4).

For children haemodialysed in an adult centre, legislation or written national recommendations require the presence of a paediatrician in 8 (22%) countries (Fig. 3 and Supplementary Table 3).

The availability of physicians and nurses to provide HD outside of regular hours (urgent HD) is required by law in 16 (44%) and 10 (28%) countries, respectively. Provision of urgent HD outside of regular hours is required by national law or written national recommendations in 14 (39%) countries (Fig. 3 and Supplementary Table 5).

## Discussion

The present study highlights the scarcity and major disparities in the laws and written recommendations governing the organisational aspects of paediatric maintenance HD in Europe. There are indeed major differences between European countries regarding the minimum legal or recommended age for paediatric patients in adult HD units, the structure of the multidisciplinary team in the units and the provision of on-call services. When they are present, the type of rules (law or written consensus) is probably a function of the national legal system, but in both cases seems to be evidence of a highly structured organisation.

Another key finding is a highly uneven distribution of centres identified as in charge of paediatric maintenance HD in Europe. The representatives of Montenegro and Cyprus declare having no HD facility for paediatric maintenance HD in their country, probably because of rarity of maintenance HD in such small countries. In other countries, the mean number of national residents below 18 years of age for whom HD has to be provided varies by a factor of 10. This heterogeneity in the density of centres is probably due to a number of factors: historical, political, geographical and financial, but mainly to the prevalence of kidney failure, transplantation, peritoneal dialysis and HD. Nevertheless, heterogeneity between countries still persists after considering differences in national HD prevalence, as shown by the estimated mean number of children receiving HD per centre. This difference in centre density also probably reflects the difficulty of defining what a dialysis centre in charge of paediatric HD is. Thus, some children are probably dialysed in adult units not considered by the national representative as facilities in charge of paediatric HD. In 7 (19%) countries. all dialysis facilities in charge of children are mixed adult–paediatric, while in 19 (53%) other countries, all or nearly all facilities are exclusively paediatric. The proportion of children treated in exclusively paediatric or mixed adult–paediatric facilities is known in some countries: around half of children in each type of facility in the United States in 2016 [6], while in France, 90% of children started their dialysis in a paediatric centre in 2021 [8]. The strengths and weaknesses of each of these two types of centres for the care of children are different; however, the outcome of children receiving HD in mixed versus dedicated paediatric dialysis centres has not been studied. Differences in reimbursement by the national health insurance system do not explain this variability in the density of centres, as shown by the total coverage of costs in all countries. While long distances to the HD centre may pose a substantial additional burden to the families, e.g. for children in a vast country like Finland, this has to be balanced against small patient numbers on HD and greater experience in larger HD centres, especially for children with rare or complex conditions.

Anatomical, (patho-) physiological and psychosocial reasons determine that HD in children differs substantially from that in adults. It is probably inadequate that 27 (75%) of the 36 European countries have no rule regarding the lowest age allowing for care in adult dialysis centres, while only 5 (14%) have a law and 4 (11%) have written recommendations by the Paediatric Nephrology Society. No limitations for age are defined in three countries, probably because all centres in charge of paediatric HD in these three countries are mixed adult–paediatric. Conversely, 12 countries require dialysis of patients below 18 years of age in paediatric dialysis centres. Among them, eight were described as having only exclusively paediatric centres in charge of paediatric maintenance HD, while representatives of Austria, Poland, Portugal and Spain indicated that some centres were mixed adult–paediatric. Even though the findings of the survey presented herein do not inform on the number of children on HD treated in adult dialysis centres in each country, they demonstrate the need for harmonisation and setting minimum standards of care for paediatric HD to prevent potential harm for paediatric patients.

Similarly, there is a major imbalance in the minimum number of staff required between the European countries and more than three-quarters of countries do not have any legal/written minimum requirement. In contrast, French law requires that the medical staff of each paediatric HD centre includes at least two paediatric nephrologists with at least 2 years’ practice in an academic paediatric nephrology centre [9]. The patient-to-physician ratio may influence the quality of care, but physician experience, consistency in the dialysis staff, as well as investment in the care of children on dialysis probably play a greater role in improving outcomes. The maximum number of patients per nurse during HD sessions is required by law or written recommendations in less than half of the countries, while it seems particularly important for quality of care. For instance, in adults, an increase in this ratio was associated with an increase in hospital-wide 30-day unplanned readmissions in the United States [10] and a higher number of HD sessions performed by a nurse per working day was associated with an increase in patient mortality [11] and a decrease of dialysis adequacy in Korea [12]. In addition, patient survival is lower among American adults [13] and children [14] in for-profit dialysis facilities than in nonprofit centres, where the patient-to-nurse ratio is lower [15]. Finally, a higher level of experience among maintenance HD nurses has been shown to be associated with better quality of care [12, 16]. The American Society of Pediatric Nephrology recommends a ratio of one to two patients per nurse during sessions, according to the child’s age and stage of development [6] and French law requires one nurse for every two children and one nurse’s aide for every four during sessions [9]. Again, national standards are needed to comprehensively define minimal requirements for qualified personnel in dialysis centres in charge of children, including the number of physicians, nurses, nurse’s aides, dietitians, psychologists, social workers, play therapists and school teachers dedicated to children on HD. In addition, human and technical resources should be defined for life-saving emergency HD services outside normal working hours.

The critical impact of adequate care is well illustrated by the essential need for optimised nutrition. Strict dietary restrictions have to be followed, which is particularly difficult in adolescents (unless intensified HD or haemodiafiltration is performed) and young children often require tube feeding. Malnutrition and obesity are both well recognised in paediatric dialysis. Addressing these issues improves growth, reduces infections, cardiovascular comorbidities and mortality and increases the likelihood of receiving a kidney transplant [17–20]. Support by specialist dieticians should be compulsory in all countries.

The long-term impact of psychosocial aspects is also paramount [21], due to the severity of their illness, but also to the density of care. As a result, anxiety or depressive disorders are frequent [22], requiring psychological or even psychiatric care. Neurological impairments are also reported, due to the underlying disease and/or intradialytic hypotensive episode-related brain injury [23–25]. The negative social impact of kidney failure in childhood persists into adulthood and results in a lower socio-economic level, higher unemployment rate, poorer social life, fewer partnerships and longer stays within the parental home compared with healthy adults [26, 27]. This should encourage psychosocial support in HD centres in charge of children.

The present study does have some limitations. The distinction between the absence of rules and an unwritten consensus is debatable and probably sometimes subjective, which is why we combined these two answers in the analysis. Furthermore, it cannot be ruled out that the answers of national representatives may have been influenced by personal opinion and practice, rather than official rules. To minimise this risk, they were nominated and/or approved by the president of their national paediatric nephrology society; it is also of note that representatives of 36 of the 42 countries provided all the requested information, making this survey representative. There is also the question of a precise definition of “a paediatric HD centre” and “a HD centre in charge of children” that can vary from one country to another and also vary over time according to needs. Furthermore, in mixed adult–paediatric centres, human resources composing the multidisciplinary team are shared for the care of both children and adults; and in exclusively paediatric centres, it is difficult to identify human resources specifically allocated to the HD unit and these resources can vary over time according to needs (i.e. the number of children on HD decreases when the number of kidney transplantations increases). In these paediatric centres, human resources can also be shared with other paediatric sub-specialities.

While it is important to consider country-specific factors such as national income, population density and geography, essential medical requirements for safe and effective dialysis that are applicable to all countries should be established. The findings reported herein may help discussions among European paediatric nephrologists, adult nephrologists and healthcare commissioners in order to optimise the resources allocated to paediatric HD in each country according to national parameters and centres’ characteristics. For countries without laws or recommendations, these should provide a basis for the establishment of an official framework; for countries with existing laws and/or national recommendations, these findings provide the opportunity to review and adapt these to international standards.

## Conclusion

Laws or written national recommendations for paediatric maintenance HD are rare in European countries and very heterogeneous when they do exist. This calls for discussion among paediatric and adult nephrologists and national and European health authorities on the organisation of safe and effective paediatric HD practices.

## Supplementary Information

Below is the link to the electronic supplementary material.

Graphical abstract (PPTX 156 KB)

Supplementary file2 (DOCX 56.0 KB)

## Data Availability

The data that support the findings of this study are available on request from the corresponding author.
