# Supplementary material for: Survey of legislative frameworks and national recommendations governing paediatric maintenance haemodialysis in Europe
Source: Pediatr Nephrol. 2025 Jan 23;40(6):2043–51. doi: 10.1007/s00467-025-06667-8 (PMC12031754; doi:10.1007/s00467-025-06667-8)
Supplement: Supplementary file 1 — Graphical abstract (PPTX 156 KB) [file 467_2025_6667_MOESM1_ESM.pptx]

## Slide 1
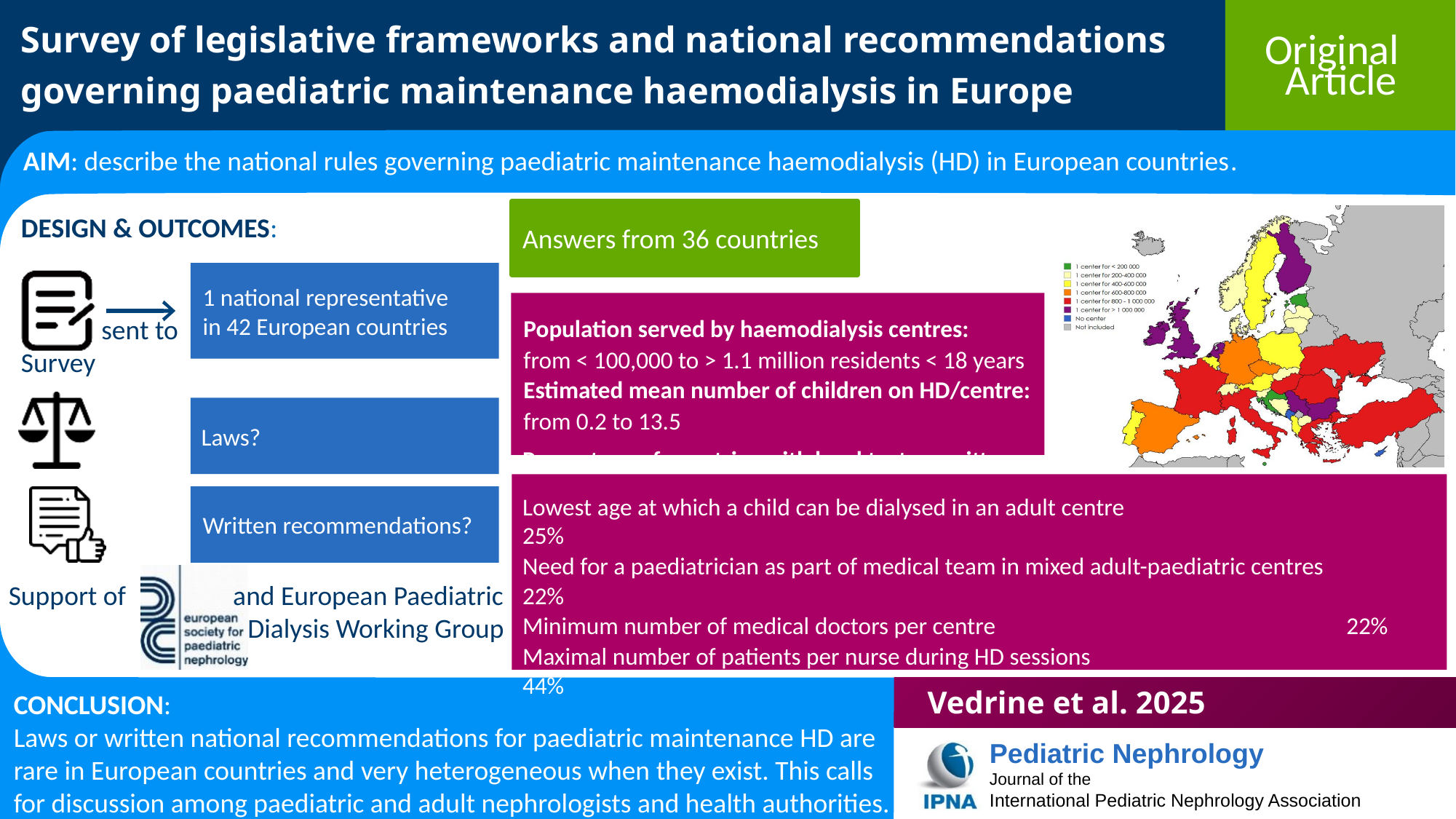

Survey of legislative frameworks and national recommendations governing paediatric maintenance haemodialysis in Europe
 AIM: describe the national rules governing paediatric maintenance haemodialysis (HD) in European countries.
Answers from 36 countries
 DESIGN & OUTCOMES:
 sent to
 Survey
Support of 	 and European Paediatric
	 Dialysis Working Group
1 national representative
in 42 European countries
Population served by haemodialysis centres:
from < 100,000 to > 1.1 million residents < 18 years
Estimated mean number of children on HD/centre:
from 0.2 to 13.5
Laws?
Percentage of countries with legal text or written national recommendations specifying:
Lowest age at which a child can be dialysed in an adult centre		 	25%
Need for a paediatrician as part of medical team in mixed adult-paediatric centres	22%
Minimum number of medical doctors per centre		 22%
Maximal number of patients per nurse during HD sessions 	 	 44%
Written recommendations?
Vedrine et al. 2025
CONCLUSION:
Laws or written national recommendations for paediatric maintenance HD are rare in European countries and very heterogeneous when they exist. This calls for discussion among paediatric and adult nephrologists and health authorities.
