# Supplementary material for: Survey of legislative frameworks and national recommendations governing paediatric maintenance haemodialysis in Europe
Source: Pediatr Nephrol. 2025 Jan 23;40(6):2043–51. doi: 10.1007/s00467-025-06667-8 (PMC12031754; doi:10.1007/s00467-025-06667-8)
Supplement: Supplementary file 2 — Supplementary file2 (DOCX 56.0 KB) [file 467_2025_6667_MOESM2_ESM.docx]

**Supplementary Table 1:** Questionnaire n°1

| **Question** | **Items** |
| --- | --- |
| Please specify your country: |  |
| Number of pediatric centers in your country doing pediatric peritoneal dialysis |  |
| Number of pediatric centers in your country doing pediatric chronic hemodialysis |  |
| Number of pediatric centers in your country doing pediatric kidney transplantation |  |
| For each centers doing pediatric haemodialysis, please, specify name, location and mail address. |  |
| What is the minimal legal age for being hemodialysed in adult units (in years) ? | <1  1  2  3  4  5  6  7  8  9  10  11  12  13  14  15  16  17  18  Not specified |
| Are the adult haemodialysis teams obliged to include paediatricians if they are treating children? | Yes  No   - Not Specified |
| What is the legal minimal MD number per pediatric hemodialysis center? | 1  2  3  4  5  Not Specified |
| What is the legal minimal MD number per pediatric PD center? | 1  2  3  4  5  Not Specified |
| What is the legal maximum ratio of patient number per nurses in a pediatric HD center during sessions? | 1  2  3  4  5  6  7  8  9  10  Not Specified |
| What is the legal maximum ratio of patient number per auxilliary nurses in a pediatric HD center during sessions ? | 1  2  3  4  5  6  7  8  9  10  Not Specified |
| Is there any legal obligation of MD on-call duty for pediatric HD? | Yes  No |
| Is there any legal obligation of MD on-call duty for pediatric PD? | Yes  No |
| Is there any legal obligation of nurse on-call duty for pediatric HD ? | Yes  No |
| Is there any legal obligation of nurse on-call duty for pediatric PD ? | Yes  No |
| Is there any obligation for availability to provide urgent HD out of the hours ? | Yes  No |
| There is a legal national regulation on the ped HD ward concerning (please select the concerned items): | Dietitian  Social Workers  In-school services  Psychologists  Play specialist,youth worker  Transition team  None  Other |
| If your answer was "other" to the precedent item, please specify : |  |
| Do you want to add some comment : |  |

**Supplementary Table 2:** Questionnaire 2

| **Question** | **Items** |
| --- | --- |
| Please specify your country: |  |
| Concerning the legal lowest age for being hemodialysed in adult units, is there in your country: | - Written legal text of the national government - Written recommendations of the national pediatric nephrology society - Unwritten consensus of the national pediatric nephrology society - None |
| Concerning the obligation for an adult haemodialysis teams to include paediatricians if they are treating children, is there in your country: | - Written legal text of the national government - Written recommendations of the national pediatric nephrology society - Unwritten consensus of the national pediatric nephrology society - None |
| Concerning the minimal MD number per pediatric hemodialysis center, is there in your country: | - Written legal text of the national government - Written recommendations of the national pediatric nephrology society - Unwritten consensus of the national pediatric nephrology society - None |
| Concerning the minimal MD number per pediatric PD center, is there in your country: | - Written legal text of the national government - Written recommendations of the national pediatric nephrology society - Unwritten consensus of the national pediatric nephrology society - None |
| Concerning the maximum ratio of children number per nurses during HD sessions, is there in your country: | - Written legal text of the national government - Written recommendations of the national pediatric nephrology society - Unwritten consensus of the national pediatric nephrology society - None |
| Concerning the maximum ratio of children number per auxilliary-nurses during HD sessions, is there in your country: | - Written legal text of the national government - Written recommendations of the national pediatric nephrology society - Unwritten consensus of the national pediatric nephrology society - None |
| Concerning the obligation of MD on-call duty for pediatric HD, is there in your country: | - Written legal text of the national government - Written recommendations of the national pediatric nephrology society - Unwritten consensus of the national pediatric nephrology society - None |
| Concerning the obligation of MD on-call duty for pediatric PD, is there in your country: | - Written legal text of the national government - Written recommendations of the national pediatric nephrology society - Unwritten consensus of the national pediatric nephrology society - None |
| Concerning the obligation of nurse on-call duty for pediatric HD, is there in your country: | - Written legal text of the national government - Written recommendations of the national pediatric nephrology society - Unwritten consensus of the national pediatric nephrology society - None |
| Concerning the obligation of nurse on-call duty for pediatric PD, is there in your country: | - Written legal text of the national government - Written recommendations of the national pediatric nephrology society - Unwritten consensus of the national pediatric nephrology society - None |
| Concerning the obligation to provide urgent HD out of the hours, is there in your country: | - Written legal text of the national government - Written recommendations of the national pediatric nephrology society - Unwritten consensus of the national pediatric nephrology society - None |
| Concerning the presence of dietitians in the paediatric HD center, is there in your country: | - Written legal text of the national government - Written recommendations of the national pediatric nephrology society - Unwritten consensus of the national pediatric nephrology society - None |
| Concerning the presence of social workers in the paediatric HD center, is there in your country: | - Written legal text of the national government - Written recommendations of the national pediatric nephrology society - Unwritten consensus of the national pediatric nephrology society - None |
| Concerning the presence of in-school services in the paediatric HD center, is there in your country: | - Written legal text of the national government - Written recommendations of the national pediatric nephrology society - Unwritten consensus of the national pediatric nephrology society - None |
| Concerning the presence of psychologists in the paediatric HD center, is there in your country: | - Written legal text of the national government - Written recommendations of the national pediatric nephrology society - Unwritten consensus of the national pediatric nephrology society - None |
| Concerning the presence of Play specialist / youth worker in the paediatric HD center, is there in your country: | - Written legal text of the national government - Written recommendations of the national pediatric nephrology society - Unwritten consensus of the national pediatric nephrology society - None |
| Concerning the presence of a transition team in the paediatric HD center, is there in your country: | - Written legal text of the national government - Written recommendations of the national pediatric nephrology society - Unwritten consensus of the national pediatric nephrology society - None |

**Supplementary table 3:** Type of rules surrounding the organisation of paediatric maintenance haemodialysis (HD) centres across Europe.

| **Country** | **Minimum age for HD in adult units** | **Obligation for adult HD teams treating children to include paediatricians** | **Minimal MD number per centre** | **Maximum number of children number per nurses during HD sessions** | **Maximum number of children number per nurse’s aide during HD sessions** |
| --- | --- | --- | --- | --- | --- |
| **Albania** | Legal text | None | None | Legal text | None |
| **Austria** | Unwritten consensus | Unwritten consensus | None | Unwritten consensus | None |
| **Belgium** | None | Legal text | None | None | None |
| **Bosnia and Herzegovina** | Unwritten consensus | Unwritten consensus | Unwritten consensus | Unwritten consensus | Unwritten consensus |
| **Bulgaria** | None | None | None | None | None |
| **Croatia** | Written recommendations | None | Legal text | Legal text | Legal text |
| **Cyprus** | None | None | None | None | None |
| **Czechia** | Unwritten consensus | None | Unwritten consensus | None | None |
| **Denmark** | Unwritten consensus | Unwritten consensus | None | Unwritten consensus | None |
| **Estonia** | Unwritten consensus | None | None | None | None |
| **Finland** | None | None | None | None | None |
| **France** | Legal text | Legal text | Legal text | Legal text | Legal text |
| **Germany** | Written recommendations | Legal text | Legal text | None | None |
| **Greece** | Legal text | None | Unwritten consensus | Unwritten consensus | Unwritten consensus |
| **Hungary** | None | None | Legal text | Legal text | None |
| **Ireland** | Unwritten consensus | None | None | Written recommendations | None |
| **Italy** | None | None | None | Written recommendations | None |
| **Latvia** | Unwritten consensus | None | None | Legal text | None |
| **Lithuania** | Unwritten consensus | Legal text | Legal text | Legal text | None |
| **Malta** | Unwritten consensus | Unwritten consensus | None | None | None |
| **Montenegro** | None | None | None | None | None |
| **North Macedonia** | Unwritten consensus | None | Unwritten consensus | Legal text | Unwritten consensus |
| **Norway** | Unwritten consensus | None | None | None | None |
| **Poland** | Legal text | None | Unwritten consensus | None | None |
| **Portugal** | Legal text | Legal text | None | Legal text | None |
| **Romania** | Unwritten consensus | Legal text | Legal text | Legal text | None |
| **Serbia** | Unwritten consensus | Unwritten consensus | None | Legal text | None |
| **Slovakia** | Written recommendations | Written recommendations | Legal text | Legal text | Legal text |
| **Slovenia** | Unwritten consensus | Unwritten consensus | None | Written recommendations | None |
| **Spain** | Unwritten consensus | None | Unwritten consensus | Unwritten consensus | None |
| **Sweden** | None | None | None | None | None |
| **Switzerland** | None | None | None | None | None |
| **The Netherlands** | None | None | None | None | None |
| **Turkey** | None | None | Unwritten consensus | None | None |
| **Ukraine** | Unwritten consensus | None | Legal text | Legal text | None |
| **United Kingdom** | Written recommendations | Written recommendations | Unwritten consensus | Written recommendations | None |

MD: medical doctor; Written recommendations: written recommendations by national paediatric nephrology society; Unwritten consensus: unwritten consensus of the national paediatric nephrology society

**Supplementary table 4.** Rules surrounding the composition of paediatric maintenance haemodialysis ward across European countries

| **Country** | **Dietitian** | **Social workers** | **In-school services** | **Psychologists** | **Play specialist / youth worker** | **Transition team** |
| --- | --- | --- | --- | --- | --- | --- |
| **Albania** | None | Unwritten consensus | None | Unwritten consensus | None | None |
| **Austria** | Written recommendations | Written recommendations | Written recommendations | Written recommendations | None | None |
| **Belgium** | Legal text | Legal text | Legal text | Legal text | None | None |
| **Bosnia and Herzegovina** | None | None | Unwritten consensus | Unwritten consensus | Unwritten consensus | Unwritten consensus |
| **Bulgaria** | None | None | None | None | None | None |
| **Croatia** | Unwritten consensus | Unwritten consensus | Unwritten consensus | Unwritten consensus | Unwritten consensus | Unwritten consensus |
| **Cyprus** | None | None | None | None | None | None |
| **Czechia** | None | None | Legal text | None | None | None |
| **Denmark** | Unwritten consensus | Unwritten consensus | Unwritten consensus | Unwritten consensus | Unwritten consensus | Unwritten consensus |
| **Estonia** | None | None | None | None | None | None |
| **Finland** | None | None | Legal text | None | None | None |
| **France** | Written recommendations | Written recommendations | Written recommendations | Written recommendations | Written recommendations | Written recommendations |
| **Germany** | Written recommendations | Legal text | Written recommendations | Legal text | None | None |
| **Greece** | Unwritten consensus | Unwritten consensus | Unwritten consensus | Unwritten consensus | Unwritten consensus | None |
| **Hungary** | Legal text | Legal text | Legal text | Legal text | None | None |
| **Ireland** | Unwritten consensus | Unwritten consensus | Legal text | None | None | None |
| **Italy** | Unwritten consensus | Unwritten consensus | Unwritten consensus | Unwritten consensus | None | Unwritten consensus |
| **Latvia** | Written recommendations | None | None | Written recommendations | Written recommendations | Written recommendations |
| **Lithuania** | Unwritten consensus | None | None | None | None | None |
| **Malta** | Unwritten consensus | Unwritten consensus | None | Unwritten consensus | None | Unwritten consensus |
| **Montenegro** | None | None | None | None | None | None |
| **North Macedonia** | None | Legal text | None | None | None | None |
| **Norway** | Unwritten consensus | Unwritten consensus | Unwritten consensus | Unwritten consensus | Unwritten consensus | Unwritten consensus |
| **Poland** | None | None | None | None | None | None |
| **Portugal** | Legal text | Legal text | Legal text | Legal text | Written recommendations | Legal text |
| **Romania** | Legal text | None | None | Legal text | None | None |
| **Serbia** | None | None | Unwritten consensus | None | None | Unwritten consensus |
| **Slovakia** | Written recommendations | Unwritten consensus | Written recommendations | Unwritten consensus | Unwritten consensus | Unwritten consensus |
| **Slovenia** | Unwritten consensus | Unwritten consensus | Unwritten consensus | Unwritten consensus | Unwritten consensus | Unwritten consensus |
| **Spain** | Unwritten consensus | None | Unwritten consensus | None | None | Unwritten consensus |
| **Sweden** | None | None | None | None | None | None |
| **Switzerland** | Written recommendations | Unwritten consensus | None | None | None | Written recommendations |
| **The Netherlands** | Written recommendations | Written recommendations | Written recommendations | Written recommendations | Written recommendations | None |
| **Turkey** | Legal text | Legal text | None | Legal text | None | None |
| **Ukraine** | None | None | Legal text | None | None | Legal text |
| **United Kingdom** | Written recommendations | Written recommendations | Written recommendations | Written recommendations | Written recommendations | Written recommendations |

**Supplementary table 5.** Rules surrounding the organisation of paediatric maintenance haemodialysis centres across Europe

| **Country** | **Obligation of MD on-call duty for paediatric haemodialysis** | **Obligation of nurse on-call duty for paediatric haemodialysis** | **Obligation to provide urgent haemodialysis out of the hours** |
| --- | --- | --- | --- |
| **Albania** | Legal text | Legal text | Legal text |
| **Austria** | Written recommendations | Written recommendations | Written recommendations |
| **Belgium** | None | None | None |
| **Bosnia and Herzegovina** | Unwritten consensus | Unwritten consensus | Unwritten consensus |
| **Bulgaria** | None | None | None |
| **Croatia** | Unwritten consensus | Unwritten consensus | Unwritten consensus |
| **Cyprus** | None | None | None |
| **Czechia** | None | None | None |
| **Denmark** | Unwritten consensus | Unwritten consensus | None |
| **Estonia** | None | None | None |
| **Finland** | None | None | None |
| **France** | Legal text | Legal text | Legal text |
| **Germany** | Legal text | Legal text | Written recommendations |
| **Greece** | Legal text | Unwritten consensus | Legal text |
| **Hungary** | Legal text | None | None |
| **Ireland** | Unwritten consensus | None | None |
| **Italy** | Legal text | Legal text | Legal text |
| **Latvia** | Legal text | None | Legal text |
| **Lithuania** | Unwritten consensus | None | Unwritten consensus |
| **Malta** | Unwritten consensus | Unwritten consensus | Unwritten consensus |
| **Montenegro** | None | None | None |
| **North Macedonia** | None | None | None |
| **Norway** | None | None | None |
| **Poland** | Legal text | Legal text | Legal text |
| **Portugal** | Legal text | Legal text | Legal text |
| **Romania** | None | None | None |
| **Serbia** | None | None | Unwritten consensus |
| **Slovakia** | Legal text | Unwritten consensus | Legal text |
| **Slovenia** | Legal text | Unwritten consensus | Legal text |
| **Spain** | Unwritten consensus | Unwritten consensus | Unwritten consensus |
| **Sweden** | None | None | None |
| **Switzerland** | Written recommendations | Written recommendations | Written recommendations |
| **The Netherlands** | Written recommendations | Unwritten consensus | None |
| **Turkey** | Written recommendations | Unwritten consensus | None |
| **Ukraine** | Legal text | Legal text | Legal text |
| **United Kingdom** | Unwritten consensus | Written recommendations | Written recommendations |

Written recommendations: written recommendations by national paediatric nephrology society; Unwritten consensus: unwritten consensus of the national paediatric nephrology society
